# ADAM17-Mediated Processing of TNF-α Expressed by Antiviral Effector CD8^+^ T Cells Is Required for Severe T-Cell-Mediated Lung Injury

**DOI:** 10.1371/journal.pone.0079340

**Published:** 2013-11-06

**Authors:** Matthew P. DeBerge, Kenneth H. Ely, Guang-Shing Cheng, Richard I. Enelow

**Affiliations:** 1 Department of Physiology and Neurobiology, Geisel School of Medicine at Dartmouth, Lebanon, New Hampshire, United States of America; 2 Department of Medicine, Geisel School of Medicine at Dartmouth, Lebanon, New Hampshire, United States of America; 3 Fred Hutchinson Cancer Research Center, University of Washington, Seattle, Washington, United States of America; 4 Department of Microbiology and Immunology, Geisel School of Medicine at Dartmouth, Lebanon, New Hampshire, United States of America; University of Iowa, United States of America

## Abstract

Influenza infection in humans evokes a potent CD8^+^ T-cell response, which is important for clearance of the virus but may also exacerbate pulmonary pathology. We have previously shown in mice that CD8^+^ T-cell expression of TNF-α is required for severe and lethal lung injury following recognition of an influenza antigen expressed by alveolar epithelial cells. Since TNF-α is first expressed as a transmembrane protein that is then proteolytically processed to release a soluble form, we sought to characterize the role of TNF-α processing in CD8^+^ T-cell-mediated injury. In this study we observed that inhibition of ADAM17-mediated processing of TNF-α by CD8^+^ T cells significantly attenuated the diffuse alveolar damage that occurs after T-cell transfer, resulting in enhanced survival. This was due in part to diminished chemokine expression, as TNF-α processing was required for lung epithelial cell expression of CXCL2 and the subsequent inflammatory infiltration. We confirmed the importance of CXCL2 expression in acute lung injury by transferring influenza-specific CD8^+^ T cells into transgenic mice lacking CXCR2. These mice exhibited reduced airway infiltration, attenuated lung injury, and enhanced survival. Theses studies describe a critical role for TNF-α processing by CD8^+^ T cells in the initiation and severity of acute lung injury, which may have important implications for limiting immunopathology during influenza infection and other human infectious or inflammatory diseases.

## Introduction

Clinical and experimental infection with influenza A virus may result in considerable lung pathology and respiratory dysfunction. While direct viral cytopathic effects can contribute to this injury, it has been postulated that an excessive or dysregulated host immune response mediates at least some of this pathology [1], [2]. CD8^+^ T cells play a critical role in the resolution and clearance of virus during influenza infection [3], [4]. However, there is also evidence that CD8^+^ T cells may contribute to immunopathology as mice deficient in T cells have significant delays in morbidity and mortality following influenza infection [5]. CD8^+^ T cells likely contribute directly to injury through cytolytic functions or indirectly through production of cytokines, such as IFN-γ and TNF-α, but it is difficult to separate the effector functions that are essential for viral clearance from those that contribute to immunopathology.

To understand the specific contribution of CD8^+^ T cells to immunopathology during influenza infection, our laboratory has used a transgenic mouse to model influenza pneumonia, while eliminating the complicating variable of direct effects of the virus infection itself. In this model, the gene encoding the hemagglutinin (HA) of A/Japan/57 H2N2 influenza A virus is expressed in alveolar type II epithelial cells under the control of the surfactant protein C (SPC) promoter. Lung injury in these SPC-HA transgenic mice is induced by adoptive transfer of HA-specific CD8^+^ T cells, which recognize an antigen corresponding to amino acids 210–219 of HA [6]. The pathology mediated by HA-specific CD8^+^ T cells in this system is severe, often lethal (depending upon the number of T cells transferred), restricted to the lung and requires expression of TNF-α by the transferred CD8^+^ T cells [7], [8]. Transfer of TNF-deficient HA-specific CD8^+^ T cells induces minimal lung injury compared to transfer of HA-specific TNF-producing CD8^+^ T cells [7]. Consistent with a role of TNF-α in inducing lung injury, SPC-HA transgenic mice deficient in either TNF receptor 1 or TNF receptor 2 demonstrate significant attenuation of lung injury following HA-specific CD8^+^ T-cell transfer [7], [9]. Furthermore, blockade of the inhibitory receptor CD94/NKG2A expressed on activated CD8^+^ T cells results in increased TNF-α production by the T cells and enhanced lung injury [10].

The pathology mediated by TNF-α in our model is mediated to a considerable degree by the induction of alveolar epithelial cell chemokines and the subsequent cellular infiltration [8]. TNF-α, signaling through the MAPK/ERK pathway, stimulated alveolar epithelial cells to produce CCL2 and CXCL2, chemoattractant molecules for macrophages and neutrophils, respectively [11]. Chemokine expression contributes to the progressive massive recruitment of host neutrophils and macrophages into the lung, that correlates with severe diffuse alveolar damage [12]. Consistent with these findings, neutralization of CCL2 results in significantly reduced parenchymal cellular infiltration in SPC-HA transgenic mice following HA-specific CD8^+^ T-cell transfer [8]. Thus, CD8^+^ T cells can indirectly mediate immunopathology in a transgenic mouse model of influenza infection by producing TNF-α upon specific antigen recognition that results in alveolar epithelial cell chemokine production and the subsequent cellular infiltration and lung injury.

Several cell types, including macrophages, T cells, and NK cells express TNF-α. It is expressed as a transmembrane protein (tmTNF-α), which is subsequently released from the membrane as a soluble protein (sTNF-α) by a proteolytic processing event known as ectodomain shedding [13], [14]. tmTNF-α and sTNF-α have been shown to have distinct and overlapping biological functions. For example, exclusive expression of non-cleavable tmTNF-α in mouse models of septic shock renders mice resistant to deleterious effects attributable to sTNF-α [15], [16]. However, tmTNF-α has been shown to provide a level of protection similar to sTNF-α during certain types of infection [15]–[18]. These studies suggest that sTNF-α and tmTNF-α mediate many of the deleterious and protective effects of TNF-α signaling, respectively.

ADAM17 (A disintegrin and metalloproteinase), also known as TNF-α converting enzyme (TACE), was identified as the primary protease responsible for proteolytic processing of TNF-α [19], [20]. ADAM17 processing of TNF-α by either leukocytes or endothelial cells has been implicated in mediating inflammation during acute lung injury [21], [22]. However, examination of the role of ADAM17 specifically in CD8^+^ T-cell effector function has been subject to limited investigation as deletion of the zinc catalytic domain of ADAM17 results in perinatal lethality [23]. Therefore, it remains unknown whether ADAM17 is responsible for processing TNF-α by effector CD8^+^ T cells, and whether proteolytic processing of tmTNF-α to sTNF-α by ADAM17 is required for severe T-cell-mediated immunopathology.

This study addresses the capacity of CD8^+^ T-cell expression of sTNF-α and tmTNF-α to initiate immunopathology in a transgenic mouse model of influenza infection. Using HA-specific CD8^+^ T cells deficient in ADAM17 activity or exclusively expressing non-cleavable tmTNF-α, we have demonstrated that ADAM17 was required for severe and lethal lung injury. However, ADAM17-deficiency did not impair CD8^+^ T-cell protection to an otherwise lethal challenge of influenza virus. Furthermore, sTNF-α, but not tmTNF-α, induced alveolar epithelial cell production of CXCL2 and the subsequent neutrophil infiltration. Consistent with these results, SPC-HA transgenic mice lacking CXCR2, the receptor for CXCL2, exhibited greatly reduced airway cellular infiltration and mortality following transfer of CD8^+^ T cells. Together, these results suggest that proteolytic processing of TNF-α by ADAM17 on CD8^+^ T cells is a critical event in CD8^+^ T-cell-mediated lung injury.

## Methods

### Ethics statement

All mice were treated humanely and all studies were conducted in accordance with guidelines approved by the Association for Assessment and Accreditation of Laboratory Animal Care (AALAC) using a protocol approved by the Institutional Animal Care and Use Committee (IACUC) at the Geisel School of Medicine at Dartmouth College. In accordance with our IACUC approved protocol, mice were euthanized via CO_2_ inhalation under anesthesia or anesthesia overdose and exsanguination. A composite endpoint was used as a surrogate for survival and mice were sacrificed, as above, either after losing ≥ 20% of the initial body weight, or other signs of severe illness, such as huddling and piloerection.

### Mice

Seven-week-old female C57BL/6, Balb/c, TNF^−/−^, CXCR2^−/−^, and Rag1^−/−^ mice were purchased from Jackson Laboratories (Bar Harbor, ME). SPC-HA transgenic mice (H-2^d^), in the Balb/c and C57BL/6 genetic background, expressing the full-length A/Japan/57 influenza HA in the distal lung were previously described and used at 7 to 12 weeks of age [6]. ADAM17^+/ΔZn^ mice expressing a deletion in the zinc catalytic domain of ADAM17, in the C57BL/6 genetic background, were kindly provided by Dr. Carl Blobel, and were bred with B10.D2 (H-2^d^ haplotype). Mice expressing a deletion of the first twelve amino acids of the TNF-α juxtamembrane extracellular domain (ΔTNF^Δ1-12^) were kindly provided by Dr. George Kollias [16]. These mice were bred with TNF^−/−^ mice which had been crossed with transgenic mice expressing ectopic H-2K^d^ to generate mice in the C57BL/6 background that exclusively express non-cleavable tmTNF-α (referred to from here on as tmTNF) [7]. CXCR2^−/−^ mice expressing the HA transgene were generated by breeding CXCR2^−/−^ mice with SPC-HA transgenic mice.

### Generation of ADAM17-deficient CD8^+^ T cells

ADAM17^+/ΔZn^ pregnant female mice were sacrificed 16-18 days after the initiation of pregnancy. Since ADAM17^ΔZn/ΔZn^ (referred to from here on as ADAM17^−/−^) mice die early in the perinatal period, homozygous knockout pups were identified by their characteristic "open-eye" phenotype at this stage of development [23], and fetal livers were harvested from ADAM17^−/−^ pups (and WT controls). Fetal liver chimeras were developed by producing a single-cell suspension from these livers and injecting them via the tail vein into sub-lethally irradiated Rag1^−/−^ mice. Six to eight weeks after transfer, immune reconstitution was confirmed, and then mice were anesthetized and intranasally infected with a sub-lethal dose of A/Japan/57 influenza virus. Three weeks later mice were sacrificed, spleens were harvested and a single-cell suspension was prepared. Splenocytes were stimulated by co-culture with irradiated Balb/c splenocytes and pulsed with HA_210−219_ peptide (a K^d^-restricted epitope of the A/Japan/57 HA) to expand and generate a bulk culture of HA-specific CD8^+^ T cells. Cells were maintained at 37°C, 5% CO_2_ in fresh Iscove’s complete media supplemented with 40 IU/ml human IL-2, and restimulated *in vitro* biweekly to expand large numbers of CD8^+^ T cells in preparation for adoptive transfer, as previously described [6], [24].

### Protection against lethal influenza infection

C57BL/6 mice received 10^ 7^ WT or ADAM17^−/−^ H-2^b^ CD8^+^ T cells specific for the H-2D^b^-restricted NP_366−374_ nucleoprotein epitope of A/PR/8/34 influenza A virus by tail vein injection. Shortly after T-cell transfer, mice were anesthetized and intranasally challenged with 10LD_50_ A/PR/8/34 influenza A virus. Mice were monitored daily for weight loss and survival.

### Adoptive transfer

HA-specific CD8^+^ T cells were removed from culture 5 days after *in vitro* restimulation and expansion, and subjected to histopaque density centrifugation to remove irradiated stimulator cells. 5×10^6^-10^7^ HA-specific cells were adoptively transferred into SPC-HA transgenic mice by tail vein injection. In some experiments, HA-specific CD8^+^ T cells were labeled with carboxyfluorescein succinimidyl ester (CFSE) prior to transfer to monitor T-cell trafficking to the lung. Mice were monitored daily for weight change and survival. Peripheral oxygen saturation was measured using the MouseOx system (Starr Life Sciences Corp., Allison Park, PA) according to the manufacturer’s protocol. Measurements of conscious mice were taken before and after T-cell transfer.

### Bronchoalveolar lavage (BAL)

At appropriate time points, mice were euthanized and tracheas were dissected and cannulated. Lungs were lavaged with 1 mL PBS as previously described [25]. The recovered fluid was centrifuged and the supernatant was frozen for later analysis of cytokines and chemokines. Cells were counted on a hemocytometer with trypan blue exclusion to determine the total number of viable cells. To determine the cell populations present, cytospin preparations were stained using the Protocol Hema 3 stain set (Fisher, Houston, TX). Neutrophils and macrophages present in BAL samples were enumerated by morphological analysis of 100 cells per cytospin preparation. The absolute number of neutrophils and macrophages was determined by multiplying the percent of cells in cytospin preparations by the total number of viable cells recovered from BAL.

### Lung histology

On day 5 after adoptive transfer of 5×10^6^ HA-specific CD8^+^ T cells, SPC-HA transgenic mice were euthanized. The trachea was dissected and cannulated and the lungs were inflated with 0.5% low melting point agarose in PBS at 25°C as previously described [26]. Inflated lungs were fixed in formalin, sectioned, and stained with hematoxylin and eosin.

### Mouse lung epithelial cell culture

Mouse alveolar epithelial-derived cells (MLE-15), stably transfected with H-2K^d^ (MLE-K^d^) were maintained as previously described [24]. Cells were plated and allowed to adhere overnight. The next day, cells were pulsed with HA_210−219_ peptide and excess peptide was removed by washing. Peptide-pulsed MLE-K^d^ cells were co-cultured with HA-specific CD8^+^ T cells for 5 hours. Supernatants were removed and stored for analysis of cytokine and chemokine production. In some experiments, 10 ng/mL recombinant mouse TNF-α (Biolegend, San Diego, CA) was added to the culture media.

### Cytokine and chemokine profile analysis

Cytokine and chemokine expression in BAL fluid recovered after CD8^+^ T-cell transfer was determined by Millipore Mouse 32-plex Luminex assay performed by DartLab (Lebanon, NH). ELISA was used to determine the albumin concentration in BAL samples (Bethyl Laboratories, Montgomery, TX). *In vitro* production of TNF-α, CCL2, and CXCL2 in co-culture experiments was determined using ELISA kits from Biolegend (San Diego, CA) and R&D Systems (Minneapolis, MN).

### Flow cytometry

The following anti-mouse monoclonal antibodies were purchased from Biolegend: FITC-CD8 (53-6.7), PerCP/Cy5.5-TNF-α (MAb11), and APC-IFN-γ (XMG1.2). Anti-CD16/32 was purchased from DartLab. For intracellular cytokine staining, HA-specific CD8^+^ T cells were stimulated with HA_210−219_ peptide in the presence of brefeldin A (10μg/ml) and maintained in brefeldin A until fixation. Cells were then blocked with anti-CD16/32 and stained with antibodies specific for CD8, TNF-α, and IFN-γ as previously described [10], and analyzed with a FACS Calibur cytometer (BD Biosciences, San Jose, CA) and FlowJo software (Tree Star, Ashland, OR).

### Statistical analyses

Statistical analysis was performed with GraphPad Prism (GraphPad Software Inc., La Jolla, CA) using a two-tailed unpaired t-test with 95% confidence interval and differences in survival after influenza challenge or adoptive transfer experiments were determined by log-rank analysis of Mantel-Cox data.

## Results

### Proteolytic processing of TNF-α by CD8^+^ T cells is required for lung epithelial cell expression of CXCL2 in vitro

**Figure 1:**
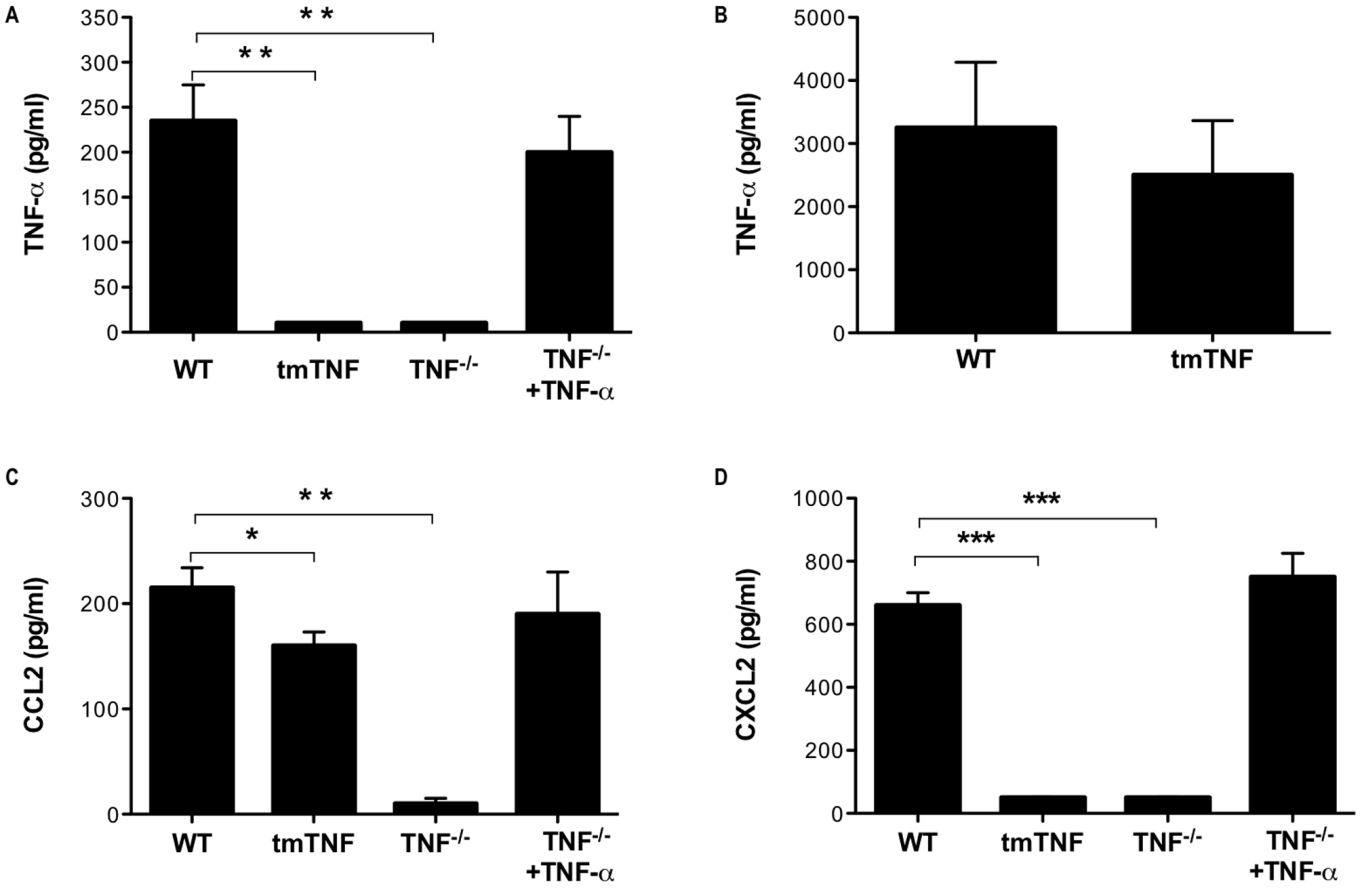
CD8^+^ T-cell processing of TNF-α is required for lung epithelial cell expression of CXCL2. MLE-K^d^ cells were pulsed with HA_210−219_ peptide and co-cultured with HA-specific CD8^+^ T cells for 5 hours. In some experiments, recombinant mouse TNF-α was added to the culture supernatant. Cell-free supernatant was analyzed for expression of (A) TNF-α, (C) CCL2, and (D) CXCL2 by ELISA. (B) Alternatively, total TNF-α production by HA-specific CD8^+^ T cells after PMA/ionomycin stimulation with protease inhibitor and detergent solubilization was measured by ELISA. Data represents mean ± standard deviation. Data are representative of three independent experiments with each condition conducted in triplicate. *P<0.05, **P<0.01, ***P<0.001.

We had previously shown that following recognition of an antigen expressed on lung epithelial cells *in vivo*, expression of TNF-α by CD8^+^ T cells was required to induce lung epithelial cell expression of CCL2 and CXCL2 [27]. We first wanted to examine whether sTNF-α or tmTNF-α expression by antigen-specific CD8^+^ T cells was required for lung epithelial cell expression of CCL2 and CXCL2. To test this, we used an *in vitro* system that reflects the interaction of CD8^+^ T cells with lung epithelial cells that occurs *in vivo*. MLE-K^d^ cells were pulsed with HA_210−219_ peptide and co-cultured with HA-specific CD8^+^ T cells that exclusively expressed non-cleavable tmTNF-α (tmTNF) and chemokine production by MLE-K^d^ cells was examined. Upon specific antigen recognition *in vitro*, tmTNF CD8^+^ T cells did not produce sTNF-α (Figure 1A). However, TNF-α production was not impaired in these cells as activated tmTNF CD8^+^ T cells expressed similar total levels of TNF-α protein compared to WT cells (Figure 1B). Next, we examined whether tmTNF-α could induce lung epithelial cell chemokine production. As expected, TNF^−/−^ CD8^+^ T cells did not induce lung epithelial cell expression of CCL2 and CXCL2 (Figures 1C, 1D). The addition of exogenous sTNF-α to co-culture of MLE-K^d^ cells and activated TNF^−/−^ CD8^+^ T cells induced MLE-K^d^ production of CCL2 and CXCL2 indicating that sTNF-α was sufficient to induce expression of these chemokines (Figures 1C, 1D). Interestingly, activated tmTNF CD8^+^ T cells were capable of inducing MLE-K^d^ cell expression of CCL2, albeit at a significantly lower level than that induced by activated WT cells (Figure 1C). However, tmTNF CD8^+^ T cells did not induce MLE-K^d^ cell expression of CXCL2 (Figure 1D). Taken together, these data indicate that proteolytic processing of TNF-α by CD8^+^ T cells is required to induce lung epithelial cell expression of CXCL2, but not CCL2, *in vitro*.

### Proteolytic processing of TNF-α is required for CD8^+^ T-cell-mediated lung injury

**Figure 2:**
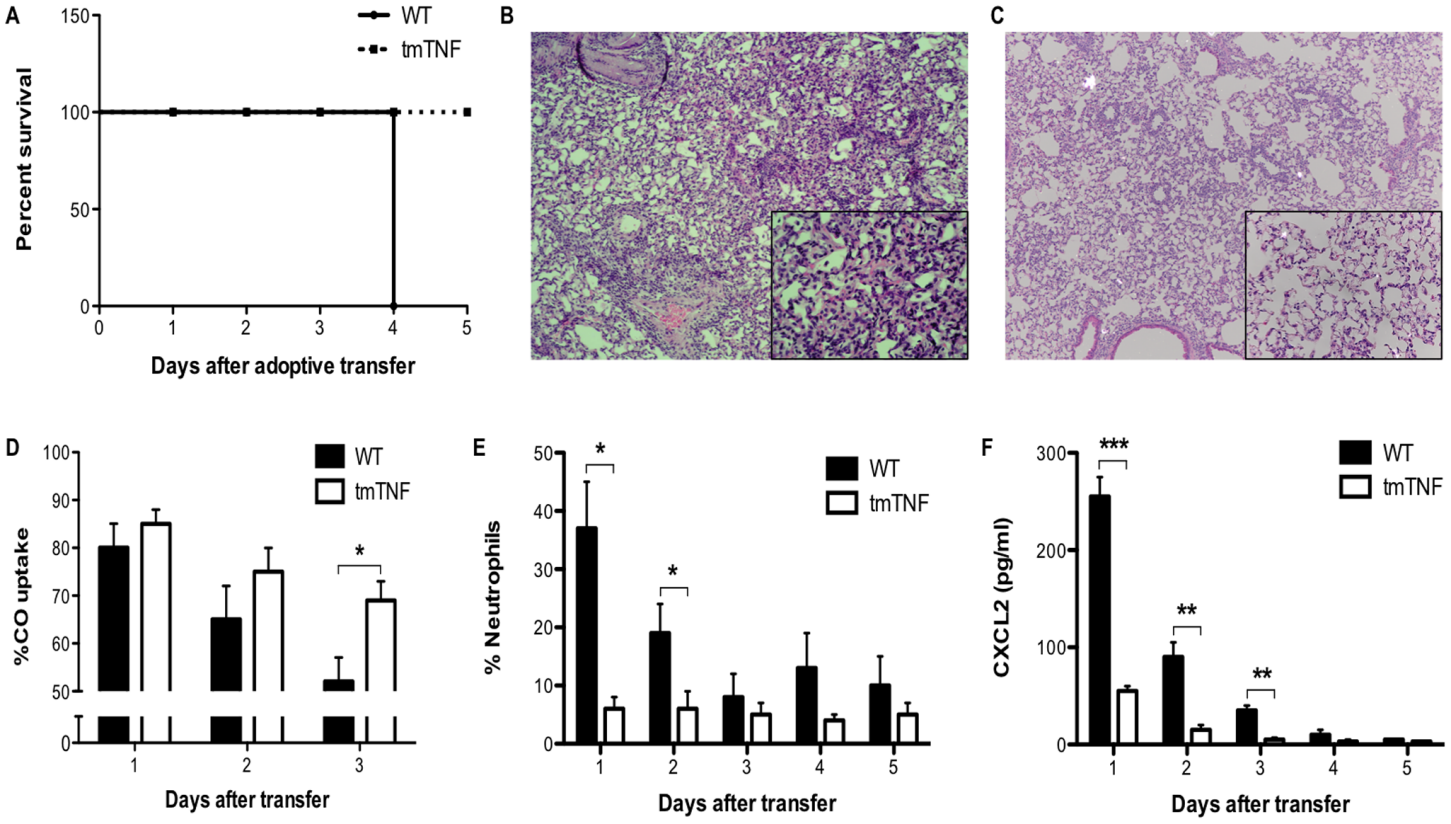
Processing of TNF-α by CD8^+^ T cells is required for severe and lethal lung injury. SPC-HA transgenic mice received WT or tmTNF HA-specific CD8^+^ T cells via tail vein injection. (A) Survival of mice after transfer of 10^7^ T cells was monitored and a significant difference in survival (*P*<0.05) was observed. Representative H&E stained lung sections from SPC-HA transgenic mice harvested 5 days after transfer of 5×10^6^ (B) WT or (C) tmTNF CD8^+^ T cells shown at 10x magnification with 40x inset. (D) Carbon monoxide uptake was measured to assess lung function. (E) Percentage of neutrophils in whole lung homogenates was determined by morphological analysis of cells after staining cytospin preparations. (F) ELISA was used to assay CXCL2 expression in cell-free whole lung homogenates. Data represent mean ± standard deviation. Data are representative of at least two independent experiments with 3-4 mice per group. *P<0.05, **P<0.01, ***P<0.001.

After demonstrating that proteolytic processing of TNF-α by CD8^+^ T cells was required for alveolar epithelial cell production of CXCL2 *in vitro*, we next examined whether proteolytic processing of TNF-α on the surface of activated CD8^+^ T cells was required for CD8^+^ T-cell-mediated lung injury *in vivo* in a non-infectious transgenic mouse model of influenza infection. In this model, we adoptively transferred HA-specific tmTNF CD8^+^ T cells into SPC-HA transgenic mice. Recipients of tmTNF CD8^+^ T cells exhibited no mortality in contrast to WT recipients, which exhibited progressive morbidity and were sacrificed 4 days after transfer (Figure 2A). In a separate set of experiments using a lower number of transferred T cells, we observed extensive infiltration of the airspaces and septae with morphologic evidence of diffuse alveolar damage, edema and hemorrhage in recipients of WT CD8^+^ T cells 5 days after transfer (Figure 2B). In striking contrast, there was minimal infiltration of the alveolar airspace and abrogation of interstitial infiltration and septal congestion in recipients of tmTNF CD8^+^ T cells (Figure 2C). Consistent with the apparent absence of significant alveolar injury in tmTNF CD8^+^ T-cell recipients, gas exchange as measured by carbon monoxide uptake (a surrogate for oxygen diffusing capacity [6]) was significantly greater 3 days after transfer compared to WT recipients (Figure 2D). To understand the mechanisms underlying the milder lung injury resulting solely from the CD8^+^ T-cell TNF-α processing defect, lungs were harvested on sequential days after transfer and digested to analyze cell populations and chemokine expression. Total cell counts were consistently lower in tmTNF CD8^+^ T-cell recipients. In addition, there was a significant reduction in the percentage of neutrophils in the lungs of tmTNF CD8^+^ T-cell recipients (Figure 2E). We examined whether a decrease in CXCL2, a neutrophil chemoattractant, was responsible for the reduced neutrophil influx, as its expression in lung epithelial cells was not induced by tmTNF-α expressed on activated CD8^+^ T cells *in vitro* (Figure 1D). We found that there was a significant reduction in CXCL2 in the lungs of tmTNF CD8^+^ T-cell recipients 24 hours after transfer that remained significantly lower 3 days after transfer compared to recipients of WT cells (Figure 2F). Thus, CD8^+^ T-cell processing of TNF-α appears critical for neutrophil influx and acute lung injury, presumably through alveolar epithelial cell expression of neutrophil chemokines, including CXCL2.

### CD8^+^ T-cell-mediated lung injury depends in part on CXCR2 signaling

**Figure 3:**
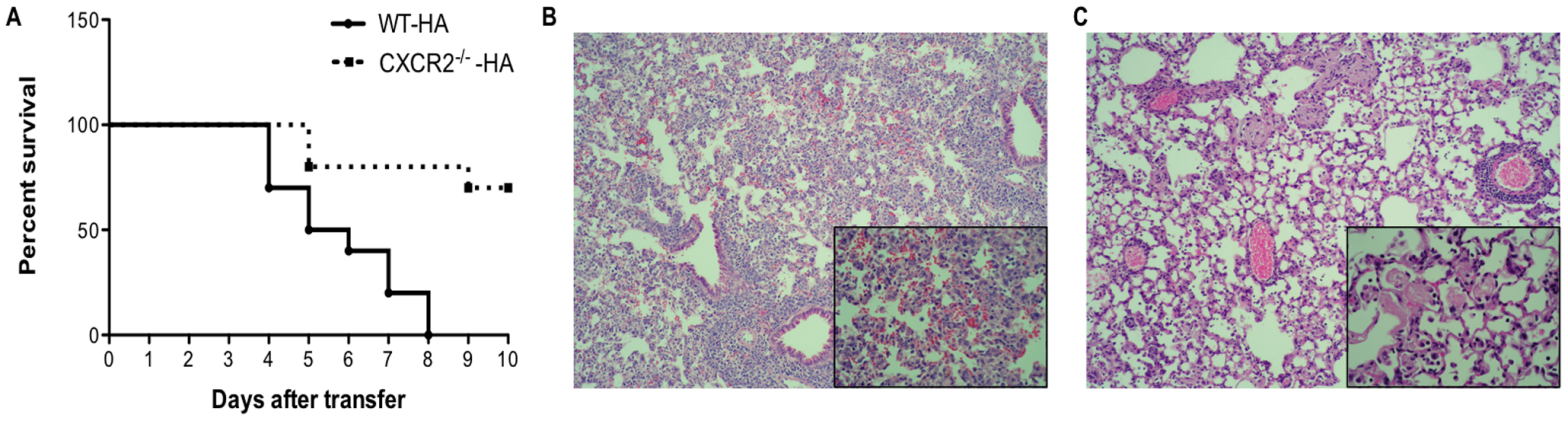
CD8^+^ T-cell-mediated acute lung injury depends in part on CXCR2 signaling. WT and CXCR2^−/−^ SPC-HA transgenic mice received 5×10^6^ WT HA-specific CD8^+^ T cells via tail vein injection. (A) Survival of mice after T-cell transfer was monitored daily and a striking difference in survival (*P*<0.001) was observed. Representative H&E stained lung sections from (B) WT-HA or (C) CXCR2^−/−^-HA mice harvested 5 days after transfer of WT HA-specific CD8^+^ T cells shown at 10x magnification with 40x inset. Data are representative of at least two independent experiments with 4-5 mice per group.

To investigate further the role of CXCL2 in mediating neutrophil infiltration into the airways and diffuse alveolar damage, we generated SPC-HA transgenic mice deficient in CXCR2, the receptor for CXCL2. Adoptive transfer of HA-specific WT CD8^+^ T cells into CXCR2^−/−^ SPC-HA transgenic mice resulted in significantly reduced mortality in contrast to WT SPC-HA transgenic animals, which exhibited progressive morbidity and eventual death of all the animals in the experiment by day 8 (Figure 3A). HA-specific WT CD8^+^ T-cell transfer into CXCR2^−/−^ SPC-HA transgenic recipients resulted in milder lung injury as shown in figures 3B and 3C. Transfer of HA-specific WT CD8^+^ T cells into WT SPC-HA transgenic recipients resulted in extensive mononuclear cell infiltration and diffuse alveolar damage with edema and hemorrhage 5 days after transfer (Figure 3B). However, there was a milder pattern of injury with diminished cellular infiltration of the alveolar airspaces of CXCR2^−/−^ SPC-HA transgenic recipients (Figure 3C). The milder injury we observed in CXCR2^−/−^ SPC-HA-transgenic animals was consistent with the increased survival of these animals following HA-specific WT CD8^+^ T-cell transfer, indicating that CXCR2 signaling is important for severe acute lung injury following CD8^+^ T-cell alveolar antigen recognition.

### ADAM17 is required for proteolytic processing of TNF-α by CD8^+^ T cells

**Figure 4:**
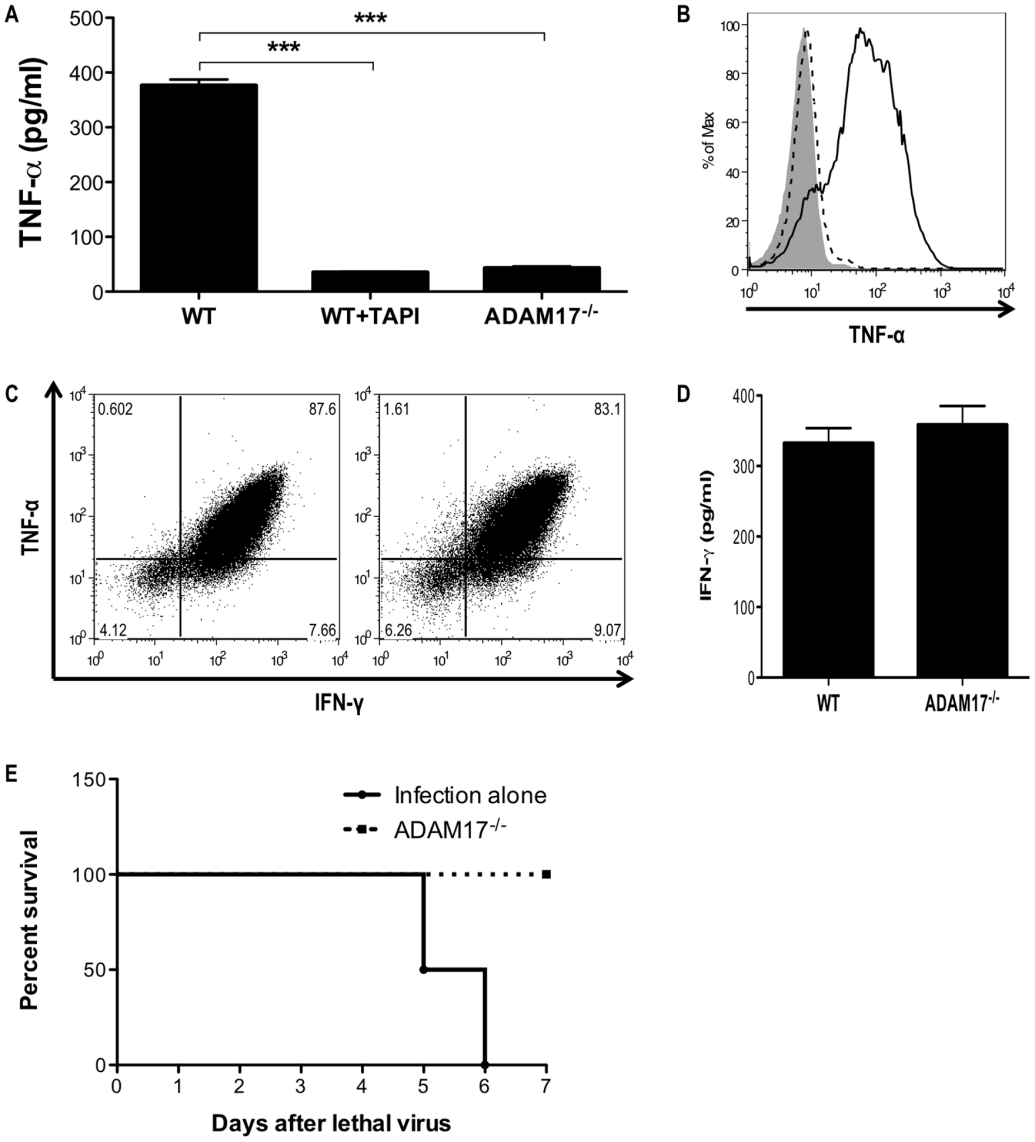
ADAM17 is required for proteolytic processing of TNF-α by CD8^+^ T cells. WT or ADAM17^−/−^ HA-specific CD8^+^ T cells were stimulated with HA_210−219_ peptide for 5 hours. (A) ELISA was used to assess soluble TNF-α production. In some experiments a TNF-α protease inhibitor (TAPI) was added at a final concentration of 50 µm. (B) Surface expression of tmTNF-α was determined by flow cytometric analysis (Ig control-Gray shading, WT-Dashed line, ADAM17^−/−^-Solid line). (C) Total intracellular production of TNF-α and IFN-γ was determined by flow cytometric analysis of T cells stimulated in the presence of brefeldin A (WT-Left, ADAM17^−/−^-Right). (D) ELISA was used to measure IFN-γ production. Data represent mean ± standard deviation. Data are representative of three independent experiments with each condition conducted in triplicate. ***P<0.001. (E) Survival of mice receiving a lethal dose of influenza A/PR/8/34 virus and media or 10^7^ NP_366−374_-specific ADAM17^−/−^ CD8^+^ T cells was monitored daily and a difference in survival (*P*<0.05) was observed. Data are representative of two independent experiments using 4 mice per group.

Since proteolytic processing of TNF-α by CD8^+^ T cells was a critical event in CD8^+^ T-cell-mediated lung injury, we investigated the cellular mechanisms regulating TNF-α processing by CD8^+^ T cells. Consistent with our previous observations, a broad-spectrum protease inhibitor blocked sTNF-α production following HA_210−219_ peptide-stimulation (Figure 4A) [9]. ADAM17 has been identified as the protease responsible for proteolytic processing of TNF-α in a number of cell types [19]. However, the role of ADAM17 in CD8^+^ T-cell processing of TNF-α and other effector functions is unknown. To examine the role of ADAM17 in CD8^+^ T-cell function, we generated HA-specific ADAM17^−/−^ CD8^+^ T cells from Rag1^−/−^ mice reconstituted with ADAM17^−/−^ fetal liver cells and immunized with influenza virus. Production of sTNF-α by HA-specific ADAM17^−/−^ CD8^+^ T cells stimulated with HA_210−219_ peptide was significantly reduced compared to stimulated WT CD8^+^ cells indicating that ADAM17 was the principal protease for TNF-α processing by CD8^+^ T cells (Figure 4A). Impaired TNF-α processing by ADAM17^−/−^ CD8^+^ T cells resulted in a significant enhancement in surface expression of tmTNF-α (Figure 4B). However, ADAM17-deficiency did not impair total TNF-α production, as ADAM17^−/−^ CD8^+^ T cells expressed similar total levels of TNF-α protein compared to WT CD8^+^ T cells when protein secretion was blocked (Figure 4C). Also, ADAM17^−/−^ CD8^+^ T cells were not deficient in IFN-γ production or secretion, suggesting that ADAM17-deficiency did not impair other effector functions (Figures 4C, 4D). Importantly, the ability to protect from an otherwise lethal influenza infection was not impaired by ADAM17-deficiency, as adoptive transfer of virus-specific ADAM17^−/−^ CD8^+^ T cells into mice infected with a lethal dose of influenza virus provided complete protection against death (Figure 4E). This indicates that ADAM17^−/−^ CD8^+^ T cells can traffic to the lung, where they appropriately recognize viral antigen, and express effector activities required for protection from lethal viral infection. Thus, ADAM17 is required for proteolytic processing and release of sTNF-α by CD8^+^ T cells, but it is not required for other CD8^+^ T-cell effector functions in virus infection.

### Expression of ADAM17 on transferred CD8^+^ T cells is required for severe and lethal lung injury

**Figure 5:**
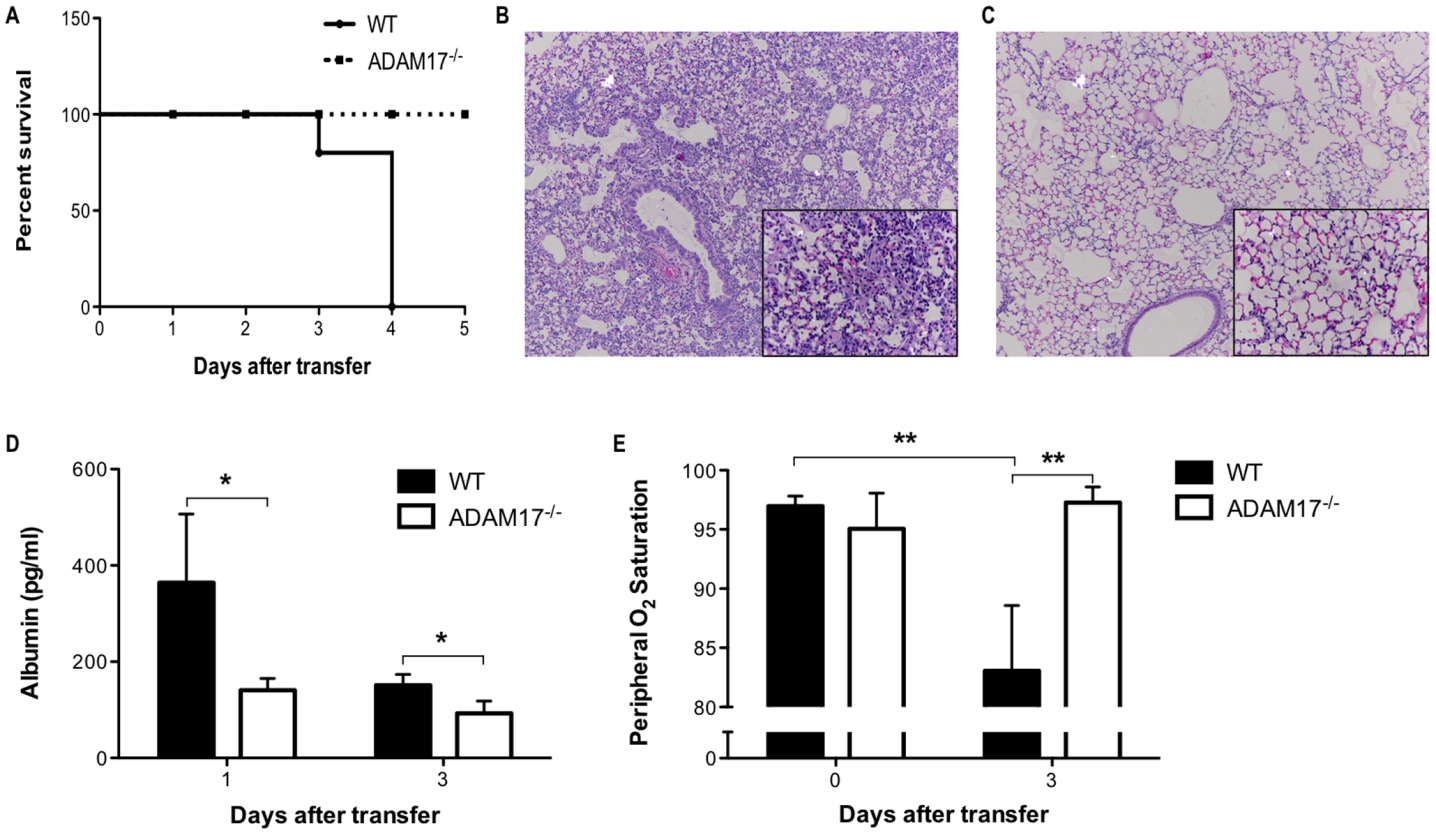
ADAM17 expression on transferred CD8^+^ T cells is required for acute lung injury. SPC-HA transgenic mice received WT or ADAM17^−/−^ HA-specific CD8^+^ T cells via tail vein injection. (A) Survival of mice after transfer of 10^7^ T cells was monitored and a difference in survival (*P*<0.05) was observed. Representative H&E stained lung sections from SPC-HA transgenic mice harvested 5 days after transfer of 5×10^6^ (B) WT or (C) ADAM17^−/−^ CD8^+^ T cells shown at 10x magnification with 40x inset. (D) ELISA was used to assess the levels of albumin in cell-free BAL fluid after transfer of 10^7^ T cells. (E) Peripheral oxygen saturation was measured in mice before and after transfer of 10^7^ T cells using the MouseOx System. Data represent mean ± standard deviation. Data are representative of at least three independent experiments with 3-4 mice per group. *P<0.05, **P<0.01.

After confirming ADAM17 as the protease responsible for processing TNF-α by CD8^+^ T cells, we investigated whether a lack of ADAM17 on CD8^+^ T cells modulated lung injury in our non-infectious, SPC-HA transgenic mouse model of influenza infection by adoptively transferring HA-specific ADAM17^−/−^ CD8^+^ T cells into SPC-HA transgenic mice. Recipients of ADAM17^−/−^ CD8^+^ T cells exhibited no mortality, in contrast to WT recipients, which exhibited progressive morbidity and were sacrificed 4 days after transfer (Figure 5A). Histologically, recipients of WT CD8^+^ T cells exhibited extensive alveolar airspace and interstitial mononuclear cell infiltration with widespread alveolar damage (Figure 5B). In contrast, ADAM17^−/−^ CD8^+^ T-cell recipients had substantially milder inflammatory cell infiltration with limited tissue damage (Figure 5C). This histological observation of milder lung injury prompted our measurement of albumin leakage into the airways, and we found that albumin in the airways of ADAM17^−/−^ CD8^+^ T-cell recipients was significantly decreased 24 hours and 3 days after T-cell transfer compared to WT recipients (Figure 5D). This suggests that there was substantially less damage to the alveolar-capillary barrier in recipients of ADAM17^−/−^ CD8^+^ T cells as presence of albumin is a marker for vascular leakage. Consistent with the reduced lung damage, no significant decrease in peripheral oxygen saturation was observed following transfer of ADAM17^−/−^ CD8^+^ T cells compared to pre-transfer measurements (Figure 5E). In contrast, there was a decrease in peripheral oxygen saturation 3 days after transfer of WT CD8^+^ T cells and this was markedly lower than the peripheral oxygen saturation observed in recipients of ADAM17^−/−^ CD8^+^ T cells 3 days after transfer (Figure 5E). Taken together, these results indicate that ADAM17 activity on transferred CD8^+^ T cells is critical for the initiation of severe and lethal lung injury in a transgenic mouse model of influenza pneumonia.

### Expression of ADAM17 is required for production of sTNF-α by transferred CD8^+^ T cells in vivo

**Figure 6:**
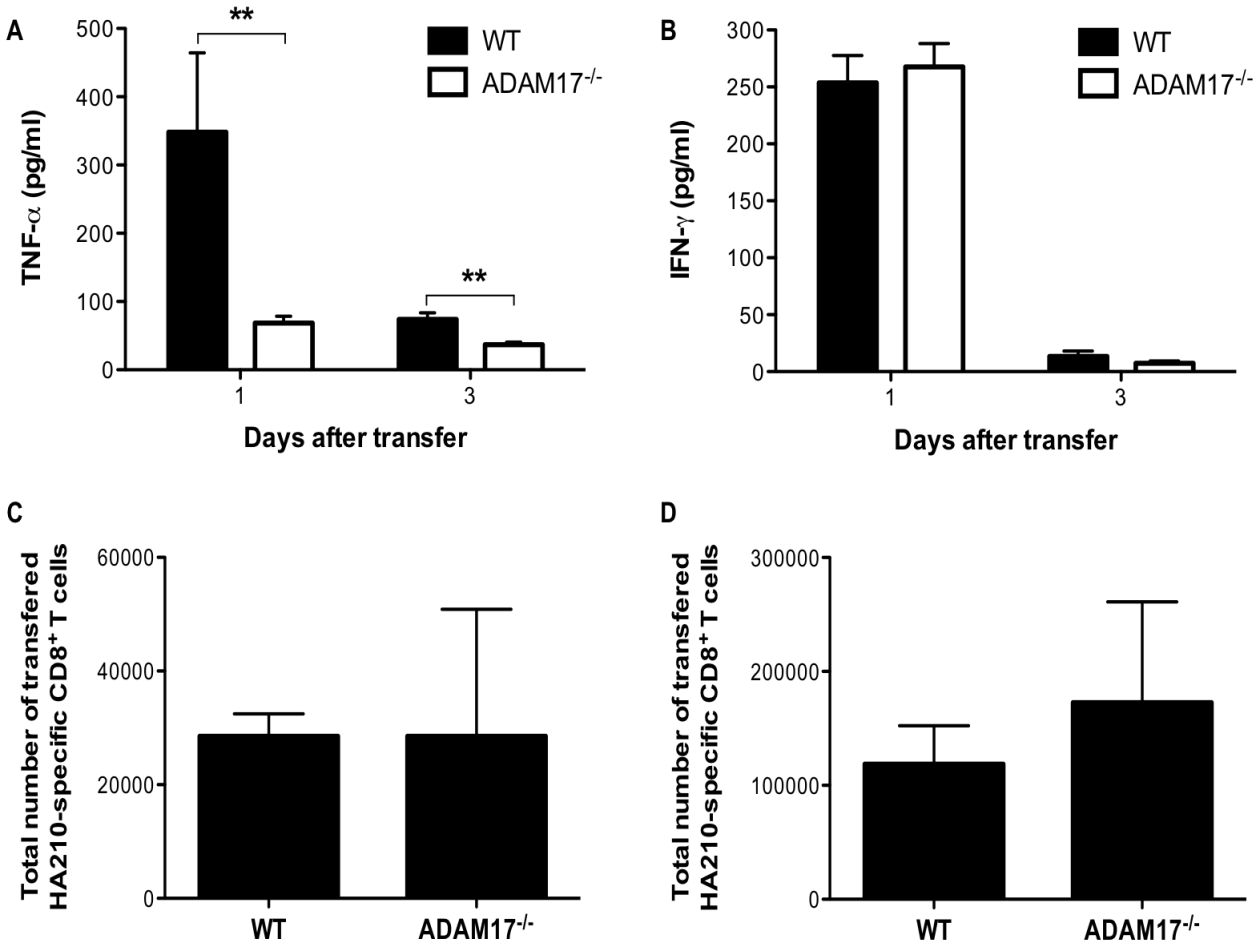
CD8^+^ T-cell expression of ADAM17 is required for TNF-α processing following antigen recognition *in vivo*. SPC-HA transgenic mice received 10^7^ WT or ADAM17^−/−^ HA-specific CD8^+^ T cells via tail vein injection. ELISA was used to assess (A) TNF-α and (B) IFN-γ expression in cell-free BAL fluid. Alternatively, HA-specific CD8^+^ T cells were labeled with CFSE prior to transfer. Twenty-four hours after transfer, cells were recovered from (C) BAL and (D) lung homogenates and assessed by flow cytometric analysis to determine the total number of transferred HA-specific CD8^+^ T cells present. Data represent mean ± standard deviation. Data are representative of two or more independent experiments with 4 mice per group. **P<0.01.

Since we observed impaired sTNF-α production following antigen stimulation of ADAM17^−/−^ CD8^+^ T cells *in vitro* and SPC-HA transgenic recipients of HA-specific CD8^+^ T cells that exclusively express non-cleavable tmTNF-α exhibit substantially milder lung injury, we speculated that the milder injury in recipients of ADAM17^−/−^ CD8^+^ T cells was due to impaired proteolytic processing of TNF-α by the transferred cells following antigen recognition *in vivo*. To assess whether ADAM17-deficiency on transferred HA-specific CD8^+^ T cells resulted in less production of sTNF-α, we recovered cell-free BAL fluid from SPC-HA transgenic mice 24 hours or 3 days after T-cell transfer and measured sTNF-α protein expression by ELISA. Following antigen recognition *in vivo*, WT CD8^+^ T cells were activated to produce and release sTNF-α, which was confirmed by the substantial amount of sTNF-α present in the airways of WT recipients (Figure 6A). In contrast, there was a significant reduction in the levels of sTNF-α on both days examined in recipients of ADAM17^−/−^ CD8^+^ T cells (Figure 6A). These results are consistent with the impaired processing of TNF-α we observe from ADAM17^−/−^ CD8^+^ T cells activated *in vitro* (Figure 4A). Furthermore, the levels of IFN-γ in the airways of WT and ADAM17^−/−^ CD8^+^ T-cell recipients were similar (Figure 6B). These results recapitulate the expression patterns of IFN-γ observed after activation of WT and ADAM17^−/−^ CD8^+^ T cells *in vitro* (Figure 4D). To confirm that similar numbers of WT and ADAM17^−/−^ CD8^+^ T cells trafficked to the lungs of SPC-HA transgenic mice, we labeled CD8^+^ T cells with CFSE prior to transfer and recovered cells from the lung 24 hours after T-cell transfer. We observed similar numbers of transferred cells in the airways and lungs of WT or ADAM17^−/−^ CD8^+^ T-cell recipients 24 hours after transfer indicating that the reduced levels of sTNF-α in the airways of ADAM17^−/−^ CD8^+^ T-cell recipients was not due to failure of the transferred cells to traffic to the lung (Figures 6C, 6D). Therefore, these data strongly suggest that both WT and ADAM17^−/−^ CD8^+^ T cells are capable of trafficking to the lung and interacting with HA antigen expressed by the alveolar epithelium. Moreover, the milder lung injury observed in ADAM17^−/−^ CD8^+^ T-cell recipients is primarily the result of impaired proteolytic processing of TNF-α.

### Expression of ADAM17 on CD8^+^ T cells is required for enhanced pulmonary infiltration of macrophages and neutrophils

**Figure 7:**
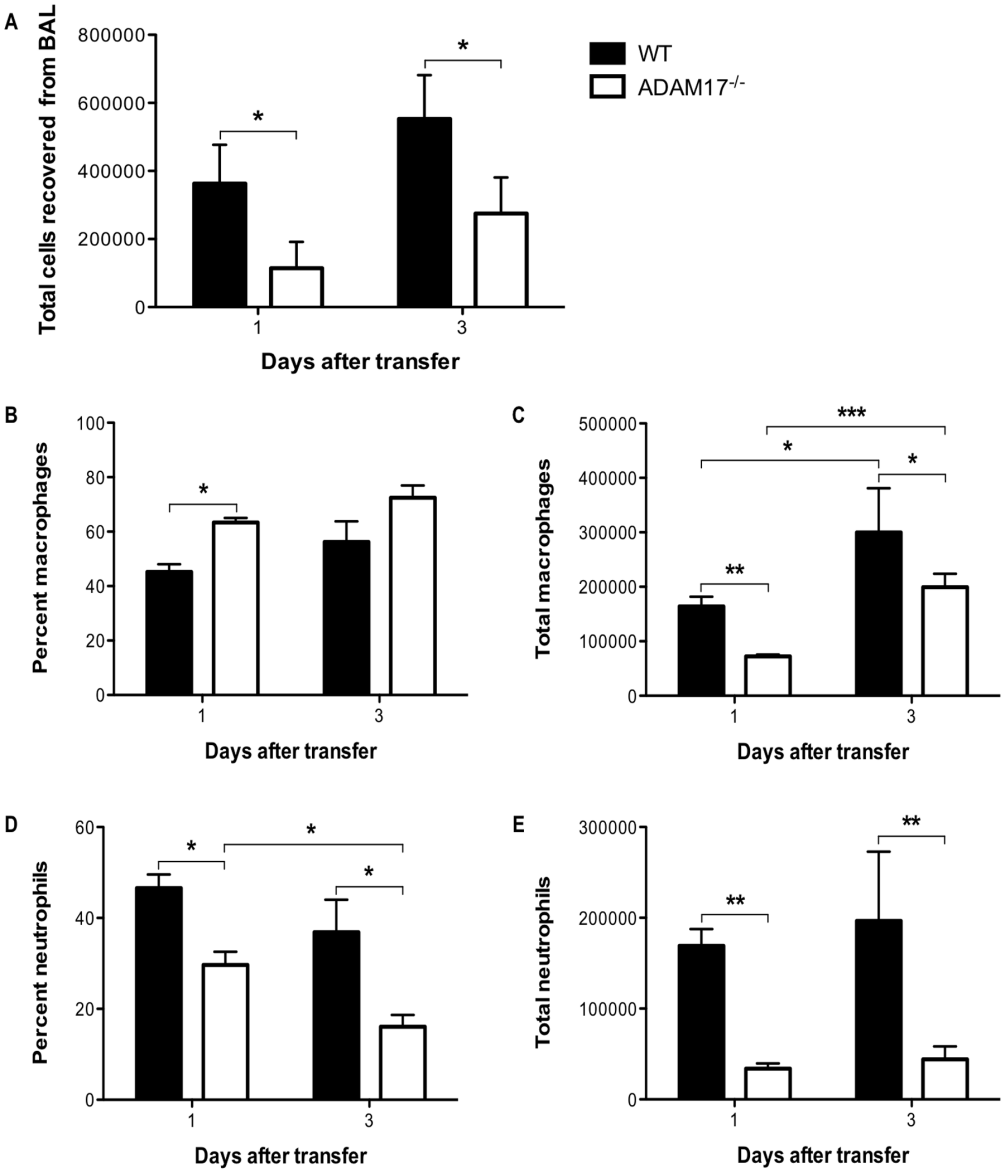
ADAM17 expression on transferred CD8^+^ T cells is required for enhanced airway infiltration. SPC-HA transgenic mice received 10^7^ WT or ADAM17^−/−^ HA-specific CD8^+^ T cells via tail vein injection. (A) On the days indicated, cells were recovered from BAL and counted on a hemocytometer with trypan blue exclusion to determine the total number of viable cells. Percentage of (B) macrophages and (D) neutrophils was determined by morphological analysis of cells after staining cytospin preparations. Absolute number of (C) macrophages and (E) neutrophils was calculated by multiplying the percent of cells by the total number of cells recovered from BAL. Data represent mean ± standard deviation. Data are representative of two or more independent experiments with 3-4 mice per group. *P<0.05, **P<0.01, ***P<0.001.

To understand the mechanisms underlying the milder lung injury resulting solely from the defect in TNF-α processing by ADAM17^−/−^ CD8^+^ T cells, we wanted to quantify the effects that transfer of ADAM17^−/−^ CD8^+^ T cells had on the pulmonary infiltration of specific inflammatory cells. We recovered cells from BAL of SPC-HA transgenic recipients at one and three days after HA-specific CD8^+^ T-cell transfer and determined the percent and absolute numbers of macrophages and neutrophils from morphological analysis of cytospin preparations. There was a reduction in the total number of cells recovered from the BAL of ADAM17^−/−^ CD8^+^ T-cell recipients on both days examined when compared to recipients of WT cells (Figure 7A). We found a slight increase in the percent of macrophages present in the airways of ADAM17^−/−^ CD8^+^ T-cell recipients 24 hours after T-cell transfer (Figure 7B). However, there was a reduction in the absolute number of macrophages in ADAM17^−/−^ CD8^+^ T-cell recipients on both one and three days after transfer when compared to recipients of WT cells (Figure 7C). There was also a reduction in the percentage of neutrophils on both days examined in ADAM17^−/−^ CD8^+^ T-cell recipients when compared to recipients of WT cells (Figure 7D). This is similar to the attenuation of early neutrophil infiltration that we observed in recipients of tmTNF CD8^+^ T cells, highlighting a critical role for early neutrophil influx and acute lung injury (Figure 2E). Additionally, there was a significant reduction in the absolute number of neutrophils on both days examined in ADAM17^−/−^ CD8^+^ T-cell recipients compared to recipients of WT cells (Figure 7E). These observations suggest that CD8^+^ T-cell proteolytic processing of TNF-α by ADAM17 exacerbates lung injury by enhancing macrophage and neutrophil infiltration of the airways.

### Expression of ADAM17 on CD8^+^ T cells is required for enhanced pulmonary inflammation

**Figure 8:**
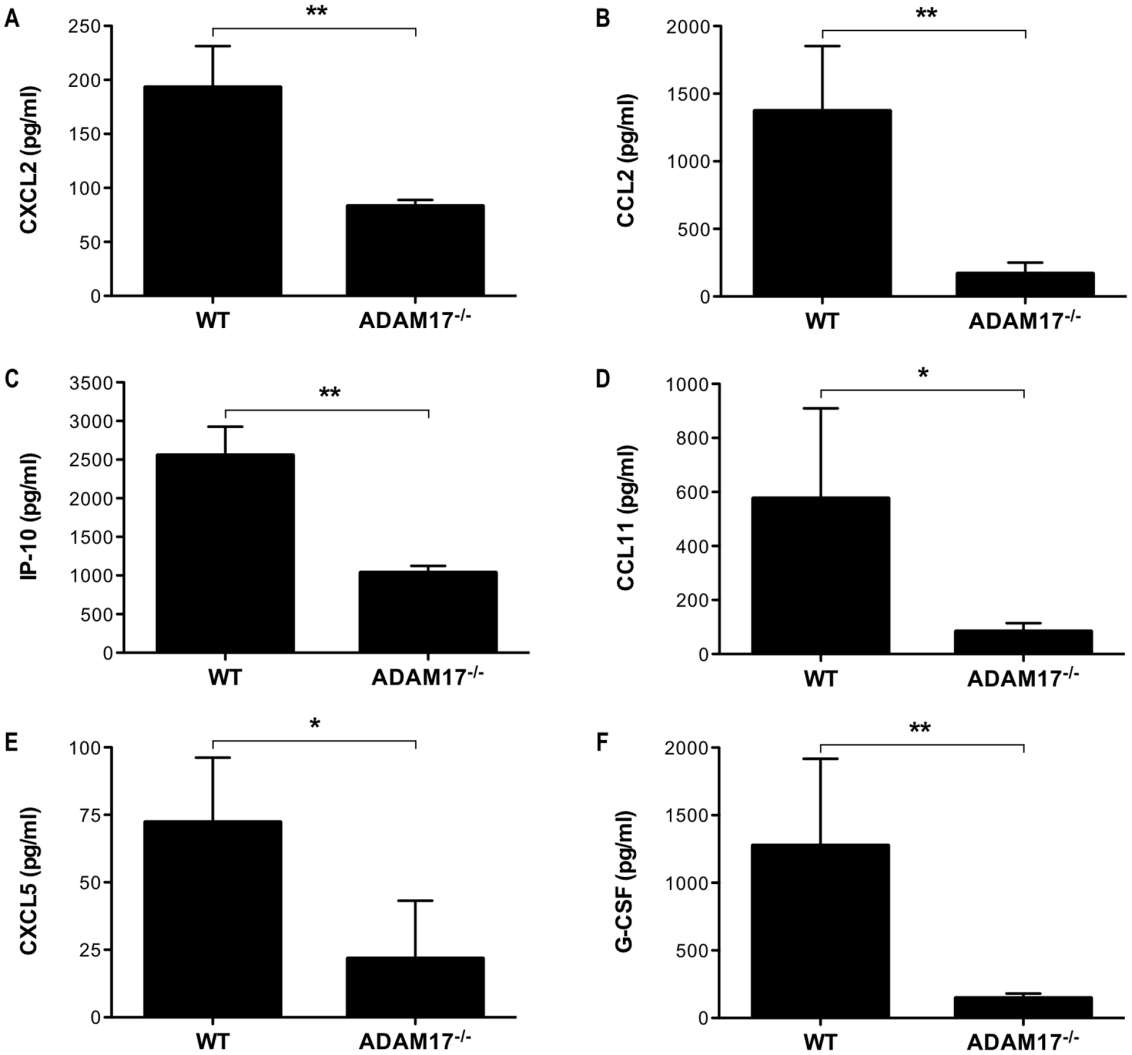
ADAM17 expression on transferred CD8^+^ T cells is required for enhanced airway inflammation. SPC-HA transgenic mice received 10^7^ WT or ADAM17^−/−^ HA-specific CD8^+^ T cells via tail vein injection. Twenty-four hours after T-cell transfer, cell-free BAL fluid was prepared and the levels of (A) CXCL2, (B) CCL2, (C) IP-10, (D) CCL11, (E) CXCL5, and (F) G-CSF were determined by Luminex assay. Data represent mean ± standard deviation. Data are representative of two mice from two independent experiments with a total of 4 mice per group. *P<0.05, **P<0.01.

With a significant decrease in pulmonary infiltration of ADAM17^−/−^ CD8^+^ T-cell recipients, we next wanted to examine the impact, which ADAM17-deficiency on transferred HA-specific CD8^+^ T cells had on lung epithelial cell chemokine expression. We prepared cell-free BAL fluid from SPC-HA transgenic mice 24 hours after HA-specific CD8^+^ T-cell transfer and measured the expression of cytokines and chemokines in the airways by Luminex assay. We observed significant differences in a variety of proinflammatory mediators with a general trend for reduced airway inflammation in recipients of ADAM17^−/−^ CD8^+^ T cells. Consistent with the decreased expression of CXCL2 observed in the lungs of tmTNF CD8^+^ T-cell recipients (Figure 2F), there was a reduction in the levels of CXCL2 in the airways of ADAM17^−/−^ CD8^+^ T-cell recipients (Figure 8A). This strongly indicates that proteolytic processing of TNF-α is required for lung epithelial cell production of CXCL2. Moreover, there was a reduction in the levels of CCL2 in ADAM17^−/−^ CD8^+^ T-cell recipients (Figure 8B). There was also a significant reduction in expression of IP-10 and CCL11 (eotaxin), chemoattractant proteins for lymphocytes and eosinophils, respectively, in recipients of ADAM17^−/−^ CD8^+^ T cells (Figures 8C, 8D). This is consistent with the observed reduction in mRNA expression of both IP-10 and CCL11 in alveolar type II epithelial cells isolated from SPC-HA transgenic mice lacking TNFR1 following CD8^+^ T-cell transfer [7]. Interestingly, we observed a reduction in CXCL5 levels in the airways of ADAM17^−/−^ CD8^+^ T-cell recipients (Figure 8E). In addition to CXCL2, CXCL5 is a neutrophil chemoattractant that mediates its effects through CXCR2. Thus, blockade of both CXCL2 and CXCL5 in CXCR2^−/−^ SPC-HA transgenic mice could contribute to the attenuated pulmonary cellular infiltrate we observed. Furthermore, there was a decrease in the expression of G-CSF, a growth factor important in neutrophil maturation, in ADAM17^−/−^ CD8^+^ T-cell recipients (Figure 8F). Using SPC-HA transgenic mice lacking Stat1, we have observed a positive correlation between G-CSF expression levels and the absolute number of neutrophils infiltrating the lungs after CD8^+^ T-cell transfer (unpublished observation). We also observed a reduction in many pro- and anti-inflammatory cytokines in recipients of ADAM17^−/−^ CD8^+^ T cells compared to WT cells, including IL-1β, IL-5, IL-6, IL-9, IL-10, IL-13, IL-15, LIF, and M-CSF (data not shown). Thus, defective TNF-α processing by ADAM17^−/−^ CD8^+^ T cells diminishes the expression of lung epithelial cell-derived chemokines and the subsequent reduction in inflammatory airway infiltration.

## Discussion

In this study, we characterized the roles of CD8^+^ T-cell expressed tmTNF-α and sTNF-α in the SPC-HA transgenic mouse model of CD8^+^ T-cell mediated immunopathology in influenza infection. The model has been designed to isolate specifically the contribution of CD8^+^ T-cell-mediated injury without the complication of viral cytopathology. We identified ADAM17 as the protease responsible for proteolytic processing of TNF-α on CD8^+^ T cells. Moreover, we observed that proteolytic processing of tmTNF-α by HA-specific CD8^+^ T cells to release sTNF-α was required to initiate severe and lethal lung injury in SPC-HA transgenic mice. Transfer of CD8^+^ T cells expressing only the non-cleavable tmTNF-α or lacking ADAM17 activity resulted in attenuated cellular infiltration and significantly milder lung injury. The reduced cellular infiltration observed in the absence of sTNF-α was due in part to the inability of CD8^+^ T-cell expression of tmTNF-α to induce lung epithelial cell chemokines such as CXCL2. We further confirmed the importance of lung epithelial cell expression of CXCL2 by transferring HA-specific CD8^+^ T cells into CXCR2-deficient SPC-HA-transgenic mice. Abrogation of CXCR2 signaling resulted in reduced airway cellular infiltration, limited impact on tissue integrity, and enhanced survival. Taken together, our study is the first to identify a critical role for ADAM17-dependent processing of TNF-α by CD8^+^ T cells in T-cell-mediated immunopathology.

As influenza HA antigen expression is widespread throughout the alveolar airspace of the SPC-HA transgenic mouse, we believe this represents a clinically challenging circumstance similar to that observed during highly pathogenic influenza virus infection, with distal airway tropism, large antigen load, and robust interference with innate immune responses. Moreover, dysregulated cytokine and chemokine expression is thought to play a role in the severe pathology associated with highly pathogenic influenza viruses. Here we show in the absence of replicating virus or innate responses, that production of sTNF-α by HA-specific CD8^+^ T cells enhanced the expression of a variety of proinflammatory cytokines and chemokines. Humans infected with the highly pathogenic H5N1 influenza virus, exhibited high serum levels of CCL2, CXCL9 and IP-10, especially in lethal cases [28]. Blockade of HA-specific CD8^+^ T-cell TNF-α processing diminished expression of these chemokines in the airways of SPC-HA transgenic mice. Furthermore, in mice infected with influenza virus expressing the HA and neuraminidase of the highly pathogenic 1918 H1N1 influenza virus, CCL2, CXCL2, IL-6 and G-CSF were found to be significantly elevated in the lungs [29]. In SPC-HA transgenic mice receiving ADAM17^−/−^ CD8^+^ T cells, the expression of these inflammatory mediators was significantly diminished. This suggests that sTNF-α production by CD8^+^ T cells might function to enhance the expression of pro-inflammatory mediators and exacerbate disease during highly pathogenic influenza infection.

Elevated levels of inflammatory mediators leading to the subsequent enhanced pulmonary infiltration as the main driver of influenza immunopathology is supported by the injury initiated by CD8^+^ T cells in this model, as diffuse alveolar damage is significantly dependent upon lung epithelial cell chemokine expression and the resulting host cellular airway infiltration. We found a direct requirement for processing of TNF-α by HA-specific CD8^+^ T cells in the induction of lung epithelial cell expression of CXCL2, as the level of this chemokine was reduced when CD8^+^ T-cell processing of TNF-α was inhibited. Furthermore, a principal role for CXCL2 in mediating lung injury was demonstrated by the observation that SPC-HA transgenic mice lacking CXCR2 displayed milder lung injury and improved survival after HA-specific CD8^+^ T-cell transfer. Other studies in mice lacking CXCR2 or CCR2, the primary receptor of CCL2, have shown that blockade of chemokine signaling reduces pulmonary recruitment and subsequently reduces injury and improves survival following influenza infection [30], [31]. In our model, processing of TNF-α by HA-specific CD8^+^ T cells is required for the enhanced recruitment of inflammatory cells to the lung, since inhibition of TNF-α processing only by the transferred CD8^+^ T cells significantly reduced the number of cells recruited to the airways. Though other cells, such as macrophages (which accumulate after initial antigen recognition by T cells) are capable of producing sTNF-α, the enhanced appearance of epithelial cell chemokines after transfer of WT CD8^+^ T cells likely results from the very early expression of sTNF-α in the hours following T-cell receptor engagement. This correlates with the early neutrophil influx observed during the first 48 hours after CD8^+^ T-cell transfer, which may set the stage for enhanced lung injury by proteolytic degradation of the basement membrane, oxidative injury of endothelial and epithelial cells, and/or production of inflammatory mediators themselves. A recent report describes early neutrophil recruitment as driving a "feed-forward" amplification of inflammation and injury in lethal influenza infection [32].

We showed previously that CD8^+^ T-cell-specific TNF-α-deficiency does not impair either virus clearance or immune protection against an otherwise lethal influenza infection, indicating that TNF-α neutralization may not compromise the antiviral response [7]. Furthermore, we found that CD8^+^ T-cell-specific inhibition of TNF-α processing did not impair host protection against influenza virus as influenza-specific ADAM17^−/−^ CD8^+^ T cells were able to provide complete protection against an otherwise lethal viral challenge. This suggests that inhibition of TNF-α processing may also lessen disease severity without compromising the immune response against the virus, and introduces a new target to limit pulmonary pathology during influenza infection. Antibody neutralization of TNF-α during influenza infection has revealed that it is possible to reduce pulmonary infiltration and pathology and improve survival without impairing virus clearance [33], [34]. Importantly however, TNF-α has been shown to play a role in protection against secondary bacterial pneumonia after influenza infection and anti-TNF-α therapies, which target both tmTNF-α and sTNF-α, have been associated with an increased risk for bacterial infection [35]-[37]. Therefore, it may be deleterious to neutralize both tmTNF-α and sTNF-α during influenza infection.

There are at least two methods in which the deleterious effects of sTNF-α can be inhibited while sparing the immuno-protective effects of tmTNF-α. The first is a class of dominant negative anti-TNF-α biological agents that selectively bind to and inhibit only sTNF-α without impairing tmTNF-α function [18]. These agents would not inhibit the proteolytic processing and release of sTNF-α by virus-specific CD8^+^ T cells, but it would block the actions of sTNF-α and may attenuate lung injury mediated by sTNF-α. An alternative approach to selectively inhibiting the actions of sTNF-α while sparing tmTNF-α might be the use of ADAM17 inhibitors, which would inhibit the proteolytic processing of TNF-α. Other studies have confirmed a role for ADAM17-dependent processing of TNF-α in acute lung injury suggesting that ADAM17 may also represent a potential therapeutic target [21], [22]. Selective inhibitors of ADAM17 have been developed, but have failed to show efficacy in treatment of rheumatoid arthritis [38]. However, two independent studies using endotoxin-triggered models of acute lung injury in mice found that intranasal delivery of selective or partially selective ADAM17 inhibitors blocked sTNF-α release and reduced vascular permeability and influx of neutrophils into the airways [22], [39]. This suggests that transient and targeted delivery of ADAM17 inhibitors can mitigate tissue injury with minimal side effects.

In conclusion, we demonstrate that ADAM17-mediated processing of tmTNF-α to sTNF-α is a critical event in CD8^+^ T cell-mediated lung injury. CD8^+^ T-cell-specific inhibition of ADAM17-mediated processing of TNF-α resulted in decreased chemokine production by alveolar epithelial cells and reduced cellular infiltration of the airways, attenuating tissue injury and mortality in a transgenic mouse model of influenza pneumonia. It remains to be seen whether differential expression patterns of tmTNF-α and sTNF-α by CD8^+^ T cells have distinct or overlapping biological functions during influenza infection and whether inhibition of ADAM17 processing of TNF-α or specific inhibition of sTNF-α can attenuate lung injury during severe influenza infection.
